# Heat Attainment and Retention in Surfers with and without a Land-Based Warm-Up and Accompanying Passive Heat Retention

**DOI:** 10.3390/sports12090241

**Published:** 2024-09-02

**Authors:** Christian J. Cook, Benjamin G. Serpell, Lauren J. Hanna, Aaron Fox, Phillip J. Fourie

**Affiliations:** 1 School of Science and Technology, University of New England, Armidale, NSW 2350, Australia; 2 Hamlyn Centre, Imperial College, London SW7 2AZ, UK; 3 Geelong Cats Football Club, Geelong, VIC 3220, Australia; 4 School of Exercise and Nutrition Sciences, Deakin University, Geelong, VIC 3220, Australia

**Keywords:** surfing, warm-up, performance

## Abstract

Surfing is a growing, high-participation recreational and competitive activity. It is relatively unique, being performed on, in, and through water with a range of temperatures. In other sports, warm-up and heat retention have proved useful at augmenting performance and ameliorating injury risk. Little work has been carried out examining this in surfing. The purpose of this work was to measure thermal profiles in surfers with and without warm-up and passive heat retention, and secondarily to assess any potential influence on free surfing. A repeated measures pre- and post- design was adopted whereby participants surfed in an artificial wave pool following an active warm-up combined with passive heat retention (experimental condition) and after no warm-up (control). Core body temperature was measured both occasions. Our results showed increases in core body temperature were greater for the experimental condition versus control (*p* = 0.006), and a time effect exists (*p* < 0.001)—in particular, a warm-up effect in the water itself was shown in both groups, possibly due to further activity (e.g., paddling) and wetsuit properties. Finally, performance trended to being superior following warm-up. We conclude that body warmth in surfers may be facilitated by an active warm-up and passive heat retention. In free surfing, this is associated with a trend towards better performance; it may also reduce injury risk.

## 1. Introduction

Surfing is a high participation and growth sport. In Australia across the 2000s, surfing participation had a growth rate of 24% year-on-year [1] and it has been reported that between 2019 and 2021 196,000 new participants registered with formal surfing clubs [2]. Furthermore, from 2016 to 2023 there was a surfing population increase of 46% amongst people aged 18 years or older [3]. Currently, in Australia, it ranks as one of the top five nature-based recreations for popularity and it is believed nearly 730,000 adults engage in surfing, spending nearly $3 billion annually. These numbers are likely to mirror world-wide numbers, where it is thought that more than 50 million people engage in surf sports [3]; however, this likely under-represents actual participation because it is a sport that men and women of all ages can participate in without engaging in organized events [1,3]. Despite the popularity of the sport, relatively little research exists surrounding performance in surfing. This is particularly surprising given surfing’s inclusion as an Olympic Sport from the 2020 Tokyo Olympics onwards. Anecdotally, surfing appears one of few sports which requires physical acts both above and below water, with padding through water, surfing on water, and disembarking the board and duck diving under water all being required. As such, data are needed to observe if other sport knowledge translates to surfing. Given the nature of the sport, aspects of warm-up and maintenance of body temperature present themselves as of interest.

Both water and wind factors affect body temperature; however, most research exploring how these elements affect body temperature has been undertaken in swimmers or divers [4,5,6,7], including a small amount with water skiers [8]. Heat loss, and heat perception, is individual in nature and it is known that for most humans water can start to markedly cool the body at 20 degrees Celsius and below [9]. Wetsuits, which are often used by surfers, are thought to ameliorate the rate of cooling; this is supported by a small amount of research conducted with surfers which showed that a 2 mm wetsuit can prevent most heat loss for short periods in water temperatures of 15 degrees Celsius or greater [10,11]. However, as wave quality and availability is often better in winter, surfers will often encounter combined chill factors well below 15 degrees Celsius. Therefore, more comprehensive and ecologically valid data examining heat attainment and retention in surfing would be useful.

From a performance perspective, core, and correspondingly muscle temperature, has an important performance-related effect—namely increasing potential power generation capacity in muscles [12,13,14] and potentially reducing injury risk [15]. For sports with relatively high muscular power demands, and in conditions where maintenance of that elevation is challenged by the environment, structuring warm-ups to include muscle temperature elevation and combining passive heat retention mechanisms to retain that heat until competition start has proved a highly successful performance strategy [16,17,18,19,20]. Even in sports where hot and humid environments may better be prepared for by pre-cooling, combinations of muscle warmth and core cooling facilitate enhanced overall performance compared to pre-cooling or warming alone [21]. In surfing competitions, one of the key judging criteria is demonstration of power on the wave and evidence shows that land-based tested strength and power increase with elite performance [22,23]. Surfing is also a timed sport, and common strategy often involves obtaining several early wave scores (the best two wave score combinations win). Therefore, having added core and muscle temperature, and potentially better early generation of muscle power, is a useful attribute. In free surfing where no judgment is applied, participants want to pop-up cleanly onto waves and catch as many good waves as possible, and they often spend maximum time on waves performing maneuvers; all of these likely have power contributions. In addition, in free surfing, reducing participation injury is a worthy goal and muscle heat may offer some amelioration for this risk [15].

The aims of this study were (1) to explore the thermal profile of surfers across the duration of a surfing session, in cold water, which would be similar in length to a competition with and without a structured warm-up combined with passive heat retention, and (2) to explore any relationships to free surfing performance (surfing in which strict competition criteria have not been demanded). We hypothesized that the warm-up and heat retention strategy would elevate core body temperature, enabling surfers to enter the water warmer and potentially sustain a higher core body temperature throughout the session. We also hypothesized that performance following the warm-up would be enhanced on waves early in the session when warm-up temperature effects were potentially more marked.

## 2. Materials and Methods

### 2.1. Study Overview

This research adopted a repeated measures pre- and post- design with randomized crossover treatment. Participants attended an artificial wave pool (UBRN, Surf, VIC, Australia) on several occasions—once for familiarization, once under experimental conditions, and once for ‘control’ conditions. For the ‘experimental’ surf, participants entered the wave pool following a combined active and passive warm-up; for the control treatment, they entered the pool without completing a warm-up. Therefore, participants acted as their own control. For both the control and experimental conditions, participants swallowed a thermometer pill 60 to 90 min prior to entering the wave pool (BMedical, Paris, France), and their core body temperature was measured every 30 s for the duration of data collection. For a subset of participants, their performance on their first two waves and their tenth wave was scored under both experimental and control conditions by two Australian state-level-qualified surfing judges (though they were not told they were being scored and they were free surfing). Only surfers who were considered ‘advanced’-level surfers (i.e., they surfers capable of executing aerial maneuvers and competed at state level in Australia) were included in this analysis because surfers of a lower standard who were recruited to this study were typically recreational surfers and inconsistent with how they approached the first two waves. We also did not tell participants they were being scored because we were interested in how they would perform on waves without the confounder of stress of competition. Consequently, core body temperature under experimental conditions was compared to core body temperature under control conditions for all participants; performance, using scoring criteria, was also compared between conditions but for a subset of participants only.

### 2.2. Participants

Thirty-four participants (n = 20 males; n = 14 females) were recruited to this study. The mean (±SD) age, height and weight for the male participants was 25.8 ± 10.6 years, 176.8 ± 7.5 cm, and 71.6 ± 10.2 kg, respectively, and for females it was 34.9 ± 11.4 years, 167.1 ± 6.1 cm, and 60.7 ± 4.4 kg, respectively. All participants ranged from being advanced surfers down to intermediate-level recreational surfers (i.e., could execute maneuvers considered one to two ‘levels’ down from advanced). Fourteen of the surfers were goofy footed (i.e., they stood on a surfboard with right foot forward), and twenty surfed with a natural stance (i.e., they stood on a surfboard with left foot forward). A total of 19 participants were included in the subset of participants who were scored by the qualified judges (15 males, 4 females). Of this subset, nine were goofy footed, and ten were natural. The mean (±SD) age, height and weight for the males in subset of the sample was 20.0 ± 6.8 years, 176.1 ± 7.7 cm, and 67.9 ± 9.1 kg, respectively, and for the females in this subset of the sample it was 32.4 ± 14.2 years, 164.5 ± 5.3 cm, and 60.0 ± 0.1 kg, respectively.

### 2.3. Procedures

Approval to conduct this research was granted by the University of New England Human Research Ethics Committee (protocol number HE22-141). Prior to commencement of data collection, voluntary informed consent to participate was provided by each participant following briefing of the research aims and protocols.

**Figure 1:**
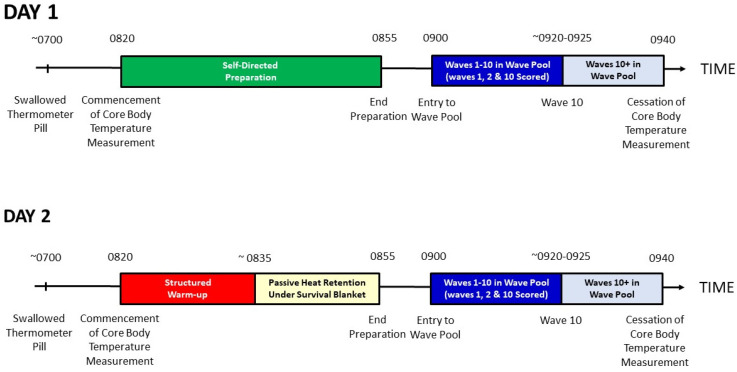
Schematic of research protocol. Note, whether participants completed session 1 or session 2 first was randomized. Both sessions were completed with more than 24 h between each other but no more than 72 h.

For familiarization, participants surfed in the artificial wave pool in their own time prior to commencement of the experiment. Thereafter, on two separate occasions, at least 24 h apart, but no more than 72 h apart, participants attended the wave pool at approximately 7:00 a.m. and swallowed an activated thermometer pill. On the control day, participants were instructed to prepare for a surf as they normally would and enter the pool at 9:00 a.m. On the experimental day, at 8:20 a.m., participants commenced a land-based warm-up in their wetsuits before covering themselves with a survival blanket until they entered the wave pool at 9:00 a.m. Survival blankets are low-weight, low-bulk blankets made of thin plastic heat-reflective sheeting. The warm-up was similar to that described elsewhere [24] and thus consisted of 2–5 min of general mobility, 2–3 min of upper body and lower body reactive strength exercises (i.e., short-contact plyometrics), 2–3 min of upper body and lower body elastic strength exercises (i.e., long-contact plyometrics), and 2–3 min of upper body and lower body mechanical power exercises (i.e., exercises which have high rate of force development predominantly concentric in nature). The warm-up took 12–15 min; therefore, participants remained under the survival blankets, remaining reasonably still, for 15–20 min. The order of days whereby participants undertook a land-based warm-up prior to entering the wave pool vs. preparing as they normally would was randomized per participant. Given that the wave pool was large and outdoors, the water temperature was not controllable, and it was reported by the pool operator to be 13 degrees Celsius at time of testing; participants wore full-length arm and leg wetsuits of at least 2 mm neoprene foam thickness. They were also instructed to wear the same wetsuit on both occasions. Core body temperature was analyzed from 8:20 a.m. to 9:40 a.m. on both the experimental and control days, and data were compared. A schematic of the research protocol can be seen in Figure 1.

### 2.4. Core Temperature Measurement

Thermometer pills were activated prior to ingestions using an eCelsius Performance Activator (BMedical, Paris, France) and swallowed by participants between 90 and 60 min prior to commencing the warm-up. As such, consistent with the literature, it was anticipated that the pills had passed through the stomach into the start of the gastrointestinal tract [25]. Participants did not eat or drink anything in the 60 min prior to ingesting the pill, and they were instructed to not consume drinks between swallowing the pill and entering the pool so as to not affect the core temperature readings while the pill was in their stomach. All temperature readings were transferred wirelessly from the participants soon after completion of the surf using an eCelcsius Performance Monitor (BMedical, Paris, France), downloaded to ePerformance Manager (BMedical, Paris, France) and exported to Microsoft Excel (version 2406) for later analysis.

### 2.5. Scoring of Performance

An advantage of using an artificial wave pool is the ability to control the ‘type’ of wave participants surfed and when they surf it. As such, the waves each participant surfed on each occasion were standardized, with the first being an ‘intermediate’-level wave, for warm up purposes, and the second and tenth waves being ‘advanced turn’-level waves. Furthermore, wave 10 was always completed approximately 20 min after wave 2 (i.e., in 20 min, participants surfed nine waves). For the intermediate-level wave, the wave face height was 1.0 to 1.5 m. For the advanced-turn wave, the wave face height was 1.6 to 2.0 m. For both wave types, the wave length was 12 to 16 s. For wave one, two and ten participants were scored for if they successfully ‘popped-up’ on the wave (yes/no). For waves two and ten only, they were also scored for quality of entry (scale of three to five with increments of 0.5 and a high score being favorable), quality of each maneuver (scale of zero to ten with increments of 0.5 and a high score being favorable), and if they completed one, two, or three maneuvers. Finally, an overall score was established across the entire wave for waves two and ten from ‘pop-up’ to exit (scale of zero to ten with increments of 0.5, with a high score being favorable). The overall score was awarded in accordance with standard Surfing Australia judging criteria [26], which was judges’ perceptions of ‘flow’, ‘speed’, ‘style’, ‘power’ and ‘progression’ (i.e., innovation and risk of maneuver on wave). Scores between judges were averaged, and when the average sat between increments of 0.5, the score was rounded up (e.g., a score of 5.75 was rounded to 6.0). Thus, scoring data were largely categorical in nature.

### 2.6. Statistical Analysis

A linear mixed model was developed with the dependent variable being the temperature change from baseline (i.e., start of warm-up), considering time (i.e., end of active warm-up, end of passive warm-up, wave pool entry, approximate time for wave 10, and end of session) and condition (i.e., control, warm-up) as fixed effects and the participant as a random effect.

For the subset of participants’ whose data were analyzed for performance, descriptive data were initially explored. Thereafter, given that the data were somewhat categorical in nature, non-parametric statistical analysis was performed to establish whether a difference existed for any scoring variables where present for entry score, maneuver scores and overall scores using a Friedman’s test. Where a significant difference was indicated, separate Wilcoxon tests were performed on all possible combinations to establish where the significant differences existed. Where significant differences existed, the Wilcoxon effect size (r) was also established.

## 3. Results

**Figure 2:**
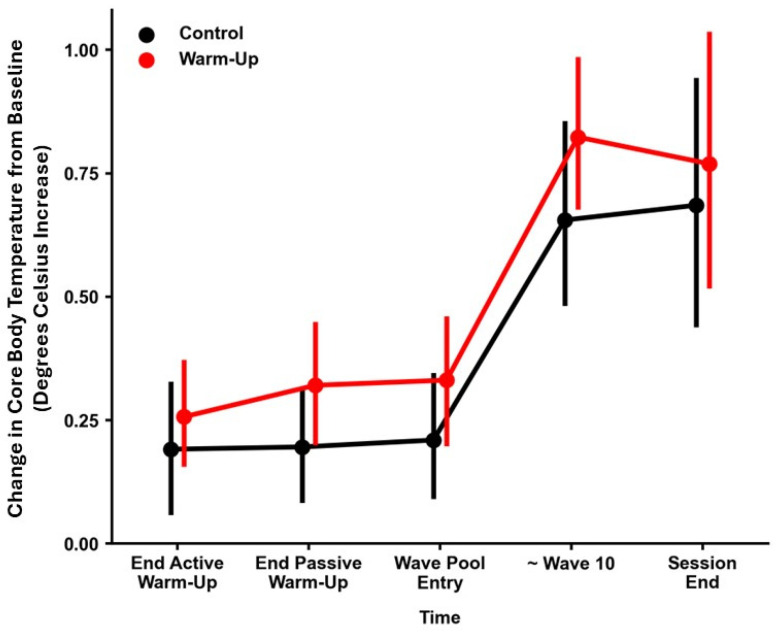
Mean core body temperature over time under control conditions vs. warm up conditions. Values are mean ± 95% confidence interval.

The baseline core body temperature was between 35.5 and 36.8 degrees Celsius. The overall performance for the model exploring core body temperature as per R-squared was 0.55. Statistically significant main effects were present for time (*p* < 0.001) and condition (*p* = 0.006), but no statistically significant interaction effects were present for time and condition. Post-hoc analysis revealed a main effect for condition where control had a lower temperature change compared to warm-up as an effect across the entire surfing session (*p* = 0.006). For the main effect of time, there were statistically significant temperature changes between the end of the active warm up, end of passive warm-up and wave pool entry to the approximate time of wave 10 and the end of the session (see Figure 2).

For scoring metrics, as can be seen in Table 1, descriptive statistics reveal less people execute maneuvers on later waves, and it appeared that participants were less likely to complete as many maneuvers as possible on later waves. Furthermore, the Friedman test showed a significant difference for ‘entry score’ (χ^2^(3) = 7.96, *p* = 0.04). Wilcoxon analyses revealed that entry score under warm up conditions for wave two was better than wave ten under warm-up conditions (*Z* = 2.11, *p* = 0.03, r = 0.48), although wave ten was not significantly different across treatments. Interestingly, the entry score for wave two was better under warm-up conditions (*Z* = 2.30, *p* = 0.02, r = 0.53). No other statistically significant observations were made, although the trends in Table 1 suggest a slight favorability across all criteria in the warm-up condition.

## 4. Discussion

The primary purpose of this work was to explore core body temperature changes in surfers with and without a structured warm-up combined with a passive heat retention strategy (wrapping post active warm-up in a survival blanket). Secondary to this, in a subset of participants who were advanced surfers, we examined whether warm-up influenced performance criteria across free surfing. Our results showed a clear thermal advantage to undertaking an active warm-up combined with a passive heat retention strategy prior to surfing. Specifically, we saw that the warm-up protocol resulted in consistently high core body temperature changes compared to baseline versus control conditions. Furthermore, this was maintained across the entire pool session. Across the session, both treatments showed further warming-up, likely a product of paddling and surfing combined with the effects of a wetsuit [27,28]. A well-designed wetsuit with a combination of 3/2 mm thickness (i.e., chest and back panels of wetsuit made of 3 mm neoprene foam, whereas limb panels are made of a 2 mm neoprene foam) will almost certainly facilitate heat retention. Finally, and secondary to the thermal effect, our data suggest that performance in free surfing may be slightly enhanced following the warm-up.

Free surfing is characterized by the surfer’s freedom to choose maneuvers, length of ride, taking risk, having fun, etc., and is not bound by any scoring criteria. However, it is possible to characterize each ride using standard performance criteria with the expectation that lower scores may be observed (participants are less concerned by trying to achieve standardized criteria). Hence, we utilized standard Surfing Australia judging criteria applied by two independent blinded judges to observe if any performance data trends emerged. At a descriptive level across all criteria, post-warmup waves scored slightly higher. In particular, pop-ups were more likely and the entry score of the pop-up on wave two was higher, as were the number of maneuvers following warm-up. In addition, variation across performance scores following warm-up was less than in the control (i.e., the interquartile range was lower under warm-up conditions versus the control). Wave ten performance was slightly less than wave two, possibly reflecting some fatigue. Certainly, catching nine waves in 20 min is likely to have been physically challenging; rarely would surfers typically catch waves with such frequency. Furthermore, in “free” surfing, surfers will often go longer on waves past the point where they receive best scores. Thus, by wave 10, fatigue may have been more than would have been observed in a directed, competitively scored situation. Speculation is difficult given the open end of what surfers perceive in free surfing; however, the results suggest that warm-up did affect aspects of performance early in the surf session. This is consistent with other data around the performance effects of warm-ups [16,21,29]. The lack of clear effect by wave ten does not negate this potential benefit as the warm-up treatment was not in any way penalized relative to the control treatment. Is it unknown if injury risk was reduced; however, as warmth has been suggested as ameliorating risk in land-based sports [15], this may be an additional potential benefit.

An extension of this work would be the converse—research in a very hot and humid environment, where surfing can also often occur. For other sports, where competition occurs in hot and humid environments, previous work has suggested precooling to be beneficial to performance [30]. However, in sports involving a benefit from early power availability, the innovative combination of both warming (legs) and cooling (torso) has been shown to offer benefits that exceed either treatment alone [21], something worthy of further surf performance research. Thus, the results from this study are certainly provocative to suggest further work should be undertaken, including under surfing competition conditions where surfers must perform to judged criteria. Additionally, our results speculatively suggest that even in recreational free surfing there may be advantages to warm-up protocols in terms of ability to catch waves, stay on waves, and execute more maneuvers (likely equating to more fun, a major goal of free recreational surfers).

Numerous other studies have shown that the use of passive heat maintenance, such as survival blankets, can maintain and even add to body heat from active warm-ups [16,31,32]; and data herein support the same in surfing. Often, the warm up needs to be separate in time from the actual competition start and in surfing anecdotally it is popular to sit and analyze waves immediately before water entry. The addition of a passive warming technology from our study appears ideal to assist in heat maintenance under such conditions. However, it is worth noting that thermal comfort and individual perception is another interesting factor which can influence clothing choice; for example, research has shown that synthetic garments enhance comfort and can sometimes trump performance-based choices in terms of adoption of a practice [33,34,35]. As such, factors other than performance gains may influence the likelihood of uptake of passive heating strategies. In terms of the outcomes speculated for free surfing and recreational surf markets, clothing designs incorporating comfort, fashion, and appropriately designed and tested warmth retention may be useful.

While this study produced some interesting findings, the results should not be considered without their limitations. The main being that thermometer pills were swallowed relatively soon to when the experimental measurements started. Research suggests that thermometer pills should be swallowed nearly six hours prior to experimental measurements so they can lodge in the intestines, not stomach, and not be subject to rapid changes in temperature due to the consumption of food and drink [25]. However, recent work has shown that, if unaffected by such confounding factors, then thermometer pills can still accurately measure core body temperature [25]. Hence, we asked participants to not eat or drink between consumption of the pill to when they entered the wave pool. Another limitation was the scoring method adopted for the overall score applied to a free surfing format; given that the overall score considered performance across the whole wave, and not all participants completed three maneuvers on each wave, a person who only completed one maneuver, for example, may have scored disproportionately high or disproportionately low, whereas a participant who completed three maneuvers on each wave is likely to have been scored more accurately, and achieving more scoreable maneuvers would be something a surfer would aim for in a competitive scenario (as opposed to maximum fun in free surfing). Nonetheless, it should be noted that for participants who executed two or more maneuvers on each wave, no statistically significant difference in performance between maneuver one, maneuver two, and maneuver three was typically observed; as such, scoring and performance were likely reasonably robust, and it may still be argued that some interesting trends were observed. It should also be noted that, despite them being lower after warm-up versus control, the relatively high interquartile ranges suggest that some may have had ‘better’ responses to the warm-up intervention than others. Across our sample, we had both gender and broad BMI differences. We did not attempt to tease these out as our study was focused on a broad sample effect, but obviously doing so in a further study could be insightful. Finally, it should be acknowledged that the frequency of waves was far greater than what would we be expected in real world environment. However, our arguments would largely be unaffected by this.

## 5. Conclusions

A land-based active warm-up combined with passive heat retention (e.g., being wrapped in a survival blanket) has a clear benefit for heat attainment and retention in surfers. Furthermore, our results suggest that surfing performance may be enhanced by this protocol and, speculatively, injury risk may decline. The outcomes of this study have implications for surfing performance in recreational and competitive settings, as well as product design (i.e., land-worn heat retaining garments, wetsuits, etc.).

## Figures and Tables

**Table 1:** Summary of surfing performance with or without a structured active warm-up with passive heating. Total number of participants = 19.

		Wave 1 (Median ± IQR) *	Wave 2 (Median ± IQR) *	Wave 10 (Median ± IQR) *
Number of Participants Who Popped-Up Onto the Wave				
	Warm-Up	19	19	19
	No Warm-Up	18	19	17
Entry Score				
	Warm-Up		3.0 ± 0.5	2.0 ± 1.0 ^†,‡^
	No Warm-Up		3.0 ± 1.0	2.0 ± 2.0
Number of Participants Who Executed One Maneuver on The Wave				
	Warm-Up		19	17
	No Warm-Up		18	12
Score for First Maneuver				
	Warm-Up		3.0 ± 1.0	2.0 ± 1.0
	No Warm-Up		3.0 ± 2.0	2.0 ± 1.0
Number of Participants Who Executed Two Maneuvers on The Wave				
	Warm-Up		14	9
	No Warm-Up		13	7
Score for Second Maneuver				
	Warm-Up		3.5 ± 2.5	2.0 ± 1.0
	No Warm-Up		3.0 ± 3.0	3.0 ± 1.0
Number of Participants Who Executed Three Maneuvers on the Wave				
	Warm-Up		8	6
	No Warm-Up		7	0
Score for Third Maneuver				
	Warm-Up		3.5 ± 2.0	2.5 ± 2.5
	No Warm-Up		2.0 ± 2.5	n/a
Overall Score				
	Warm-Up		4.0 ± 3.0	3.0 ± 1.0
	No Warm-Up		3.0 ± 2.0	3.0 ± 2.0

NB. * = only shown where we are not presenting the count of the variable (e.g., we are not presenting median ± IQR when showing the number/count of people popping up onto the wave). n/a = no participants surfed this wave. ^†^ = significantly lower than score on wave 2 under warm-up conditions. ^‡^ = significantly lower than score on wave 2 when no warm-up was completed. Effect size > 0.48.

## Data Availability

Data for this study are not publicly available due to guidelines from the approving institutional review board.
